# Suicidal ideation and self-injury in LGB youth: a longitudinal study from urban Switzerland

**DOI:** 10.1186/s13034-022-00450-5

**Published:** 2022-03-14

**Authors:** David Garcia Nuñez, Nesrin Raible-Destan, Urs Hepp, Stephan Kupferschmid, Denis Ribeaud, Annekatrin Steinhoff, Lilly Shanahan, Manuel Eisner, Niklaus Stulz

**Affiliations:** 1 Center for Gender Variance, Department of Plastic, Reconstructive, Aesthetic and Hand Surgery, University Hospital Basel, Basel, Switzerland; 2 Department of Consultation-Liaison Psychiatry and Psychosomatic Medicine , University Hospital Zurich, Zurich, Switzerland; 3 Integrated Psychiatric Services Winterthur – Zurcher Unterland, P.O. Box 144, CH-8408 Winterthur, Switzerland; 4 Jacobs Center for Productive Youth Development, University of Zurich, Zurich, Switzerland; 5 Department of Psychology, University of Zurich, Zurich, Switzerland; 6 Institute of Criminology, University of Cambridge, Cambridge, UK

**Keywords:** Adolescence, Homosexuality, Bisexuality, Minority stress, Self-injury, Sexual orientation, Suicidal ideation

## Abstract

**Background:**

Adolescent suicidality, suicidal ideation (SUI) and self-harming behaviour (SI) are major public health issues. One group of adolescents known to be particularly prone to suicidality and mental health problems is lesbian, gay and bisexual (LGB) youth. Although the social acceptance of the LGB community has increased in recent years, LGB individuals are still at risk of mental health issues and suicidal behaviour. More longitudinal research looking into the associations between sexual orientation (SO) and facets of mental health across adolescence is warranted.

**Methods:**

This research examined associations between sexual orientation, suicidal ideation and self-injury at 15, 17 and 20 years of age in a community-based sample of 1108 Swiss adolescents (51.1% females/48.9% males). At the age of 15 years, participants provided information regarding their SUI and SI. At 17 and 20 years of age, participants also reported their SO.

**Results:**

Twelve percent of the female participants and 4.4% of the male participants reported identifying as LGB at 17 and 20 years of age. Self-reports of bi- or same-sex attraction increased over time in both genders, with the increase being more pronounced in females. LGB adolescents of both genders showed significantly higher percentages of SUI and SI at the ages of 17 and 20 years than their heterosexual peers.

**Conclusions:**

The findings confirm a higher risk of SUI and SI in adolescents who identify as LGB. Future studies should develop interventions targeting mental health from early adolescence with the aim of reducing disparities related to SO.

## Background

In its broadest definition, sexual orientation (SO) encompasses emotional, cognitive, and behavioural dimensions [1]. While there is significant variability in the frequency of sexual identities, the prevalence of sexual behaviour and attraction does not show meaningful differences in intercultural comparisons [2]. In a recent survey from Switzerland, 4.7% (7% of females and 2.4% of males) at age 15 reported not being exclusively attracted to the opposite gender [3].

In the past few decades, social change has taken place in many Western societies, leading to a greater social acceptance of sexual minorities such as the lesbian, gay, and bisexual (LGB) communities [4]. For example, the number of people in the UK who describe same-sex sexual relationships as "always wrong" has dropped dramatically from 64% in 1987 to 22% in 2012 [5]. In parallel, research shows that attention to LGB people and LGB topics in the news has increased significantly over time [6]. In some countries, this greater acceptance has contributed to the establishment of LGB rights [7]. A multivariate, multi-level analysis shows that attitudes towards homosexuality and gay rights are shaped by both, individual- and country-level variables. Individual-level predictors such as female gender, younger age, higher education, not being an immigrant to one’s residing country, liberal political affiliation, and low religiosity, were significantly linked with more positive attitudes towards homosexuality. At the country level, a high emphasis on social conservatism, less economic development, a Communist past, and fewer civil rights for homosexuals were connected with more unfavourable attitudes [8]. However, the positive interaction between acceptance and the introduction of LGB rights is far from universal and mostly moderate in magnitude [7].

### LGB health disparities & Sexual Minority Stress Model

The sexual minority stress model (SMSM) [9] states that the unique social rejection resulting from a pervasive homo- and biphobic culture causes stress among sexual minorities, leading to long-term negative health effects. According to the SMSM, sexual minority stress can be caused by external (distal) factors such as institutionalized prejudice and interpersonal discrimination as well as by internal (proximal) factors such as internalized homophobia (IH) [9]. In fact, LGB populations are at an elevated risk for structural stigma [10] and victimization [11]. Moreover, path analyses revealed that in LGB youth, higher levels of IH and lower social support mediated the association between past parental rejection following SO disclosure and current psychological distress [12].

The SMSM provides a plausible explanation of how adverse social circumstances can have negative effects on health by means of biological and psychological mechanisms. Likewise, it can be a useful tool to address intragroup differences arising from the fact that not every (sexual) minority is exposed to the same social exclusion mechanisms [9]. Within the LGB population, for example, bisexual individuals appear to be particularly vulnerable [13].

### LGB youth & suicidality

Suicidal ideation (SUI) is very common in adolescents [14] and, in many cases, precedes suicidal behaviour and suicide. While SUI has a significant predictive value on the occurrence of suicide at a population level [15], this relationship is not generally present when considering the individual case [16]. The self-injuries that arise from SUI can aim at the death of the person concerned [17], but self-injuries can also comprise a direct and deliberate destruction of one's own body tissue without any suicidal intent [18]. Studies suggest that such nonsuicidal self-injuries (NSSI) are more prevalent in adolescents than in adult samples [19].

Differences between LGB and heterosexual youth are also evident in the prevalence of SUI and self-injury. In a meta-analytic review examining 19 studies on suicidality disparities between sexual minority and heterosexual youth (N = 122,955 persons; age ≤ 18 years), on average, 28% of LGB adolescents reported a history of suicidality (SUI, plans and attempts) in comparison to 12% in the sample of heterosexual youth [20]. This difference increased with growing severity of suicidality. Whereas LGB youth were almost twice as likely to report SUI (OR = 1.96) and suicidal plans (OR = 2.20), the odds ratio increased more than threefold for suicide attempts (OR = 3.18) and even fourfold when asked about suicide attempts requiring medical attention (OR = 4.17). Another meta-analysis on the prevalence of NSSI came to a similar conclusion: In the 15 studies examined (N = 68,848), LGB individuals showed a threefold increased risk of having carried out NSSI (OR = 3.00), with LGB *adolescents* showing an even higher risk [21]. Data from a comparison of various cross-sectional studies from Switzerland show that of all age groups (14 to 83 years of age), men under 25 years reported the highest prevalences (35.4% SUI and 11.5% attempted suicide) in the past 12 months [22].

However, the cross-sectional design of many data collections makes it impossible to track the developing SO and its influence on the trajectory of suicidality reported by study participants. Accordingly, there is a need for studies that examine individual pathways of the development of mental health problems over time. Such an approach could help clarify the question of whether problems persist and may even become more severe in LGB youth as they advance into young adulthood.

### Suicidality within LGB youth subgroups

Group comparisons within LGB youth consistently point out that bisexual youth show higher scores on SUI [20, 23] and self-injury (SI), whether suicidal [20] or nonsuicidal [21] in intent. In contrast, studies do not provide a clear picture regarding potential gender differences (lesbian/bisexual women vs. gay/bisexual men) in LGB youth. Some studies show the “gender paradox” known from the general population [23], which consists of the fact that adult women report more SUI than adult men [23], while adult men show higher lifetime suicide attempt rates than adult women [24]. However, other reviews point to the opposite [25] and observe a greater disparity in suicide risk for bisexual women (OR range: 1.48–1.95) than for bisexual men (OR range: 1.00–1.48). Again, there are also data in which no significant moderation effect for gender resulted [26]. Knowing the importance of sex and gender on socialization and stigma experiences [27] as well as on SO development [28], the recommendations indicate that more evidence is needed in this area.

### Aims of the study

Given the above evidence and the lack of studies in Switzerland on mental health in LGB youth, the main aim of this study was the longitudinal assessment of some suicidality parameters (suicidal ideation and self-injury) and sexual orientation in a nonclinical sample at two time points across adolescence (17 and 20 years of age). Based on current research findings, it was hypothesized that LGB youth would score higher on suicidal ideation and report more self-injury than their heterosexual peers at both examination times. Furthermore, an additional retrospective analysis on the association between sexual orientation (SO) at 17 and 20 years and reports of suicidal ideation (SUI) and self-injury (SI) at the age of 15 years was run. The goal of this retrospective analysis was to strengthen potential findings regarding the relationship between SO, SUI and SI across adolescence. Another aim was the exploratory investigation of potential connections between gender, SO, SUI and SI. In view of the ambiguous statements in the literature in this regard, no hypothesis was formulated, thus allowing us to interpret the results as openly as possible.

## Methods

### Sample

The sample of adolescents used in the analyses was derived from the ongoing Zurich Project on the Social Development from Childhood to Adulthood (z-proso) [29–31]. The target cohort consisted of 1675 children (48.1% females) who entered first grade in 2004 (approximately aged 7) in one of 56 public primary schools in the city of Zurich, Switzerland. The initial target sample of schools was selected using a cluster-stratified random sampling procedure. The resulting sample of children was largely representative of the city's youth population and of the number of immigrants living in Zurich, resembling a proper community sample. Ribeaud et al. provided more details on the cohort profile of the z-proso project in their recently published article [32].

Participants have been regularly followed since their school started in 2004. Data collection waves with the participating children took place at the ages of 7, 8, 9, 11, 13, 15, 17 and 20 years. Data collected in the latest three waves (2013, 2015 and 2018) of the longitudinal z-proso project were used in the present analyses, with participants currently being in their early twenties. The sample of the present research consisted of *n* = 1108 participants (66% of the target sample), for which data were available for the variables of interest at all three measurement waves under study. A total of *n* = 567 participants (42.2% females) from the recruitment sample (N = 1675) were excluded from the current analytic sample due to missing data on at least one of the variables of interest (sexual orientation, suicidal ideation or self-injury) in the three waves under study. A total of 51.1% of the analytic sample was females, and the mean ages at the three time points were 15.4 (*SD* = 0.36), 17.4 (*SD* = 0.38) and 20.6 years (*SD* = 0.38).

### Data collection

In the surveys at ages 15 and 17, data were collected by means of paper and pencil questionnaires completed by the participants in classroom settings in convenience groups during leisure time after school. The groups consisted of approximately 5 to 20 individuals who were supervised by 1 to 3 research assistants from the z-proso project team. Questionnaires were in German and completion took approximately 90 min with a short break after 45 min. At the latest measurement wave (20 years of age), participants completed a computer-aided self-administered lab-based survey entailing the same questions on sexual orientation, suicidal ideation and self-injury as in the previous wave. Adolescents and young adults received a participation incentive of 50 CHF at age 15, 60 CHF at age 17 and 75 CHF at age 20.

The study was conducted in accordance with national and international ethical standards and was approved by the responsible ethics committee at the Faculty of Arts and Social Sciences, University of Zurich, Switzerland. Adolescents provided written informed consent at each wave; until age 15, parents could opt their child out of the study.

### Measures

#### Sexual orientation (SO)

SO was assessed by means of sexual attraction at the age of 17 and 20 years but not at the age of 15 years. The scale was taken from the Zurich Youth Survey [33]. Participants were asked the following question: *"People differ in the sexual attraction that they feel towards others. How would you describe your sexual orientation? Please indicate the statement that best describes you. With “men”, we also mean “boys”, with “women”, we also mean “girls”. Please only mark one answer"*. Individuals provided their ratings on a 5-point Likert scale with the following options: I’m attracted only to men/I’m attracted mainly to men but sometimes also to women/I’m attracted equally to men and women/I’m attracted mainly to women but sometimes also to men/I’m attracted only to women. In combination with the information asked about the gender of the subjects (girl/boy), answers were dichotomized (0 = exclusively heterosexual orientation/1 = LGB orientation) for analyses. The number of participants stating an exclusively same-sex attraction was too small to be included as a separate group in the analyses (*n* = 6 at 17 years of age and *n* = 14 at 20 years of age for both genders).

#### Suicidal ideation (SUI)

One question was used to assess SUI at the ages of 15, 17 and 20 years. This question was developed especially for the z-proso project and was part of the questionnaire section on "how you feel". It was introduced as follows: *Sometimes people think about things they would never actually do. How about you? Please indicate how often you thought about these things in the last month.* Subjects were asked to provide a rating for suicidal ideation in the past month (*I thought about killing myself)* using a 5-point Likert Scale ranging from *Never* to *Very Often*. For the purpose of the current research aims, answers were dichotomized (0 = never/1 = ever).

#### Self-injury (SI)

Self-harming behaviour was assessed at the three ages under study (15, 17 and 20 years) by asking the following question: *"I harmed myself on purpose (e.g., cut my arm, tore wounds open, hit my head, tore out my hair)".* This question was developed especially for the z-proso project and was part of the questionnaire section on "how you feel" [34]. Again, participants rated their last month behaviour on the same 5-point Likert scale used for SUI. Answers were dichotomized (0 = never/1 = ever) for the purpose of the current research aims.

### Statistical analyses

**Fig. 1:**
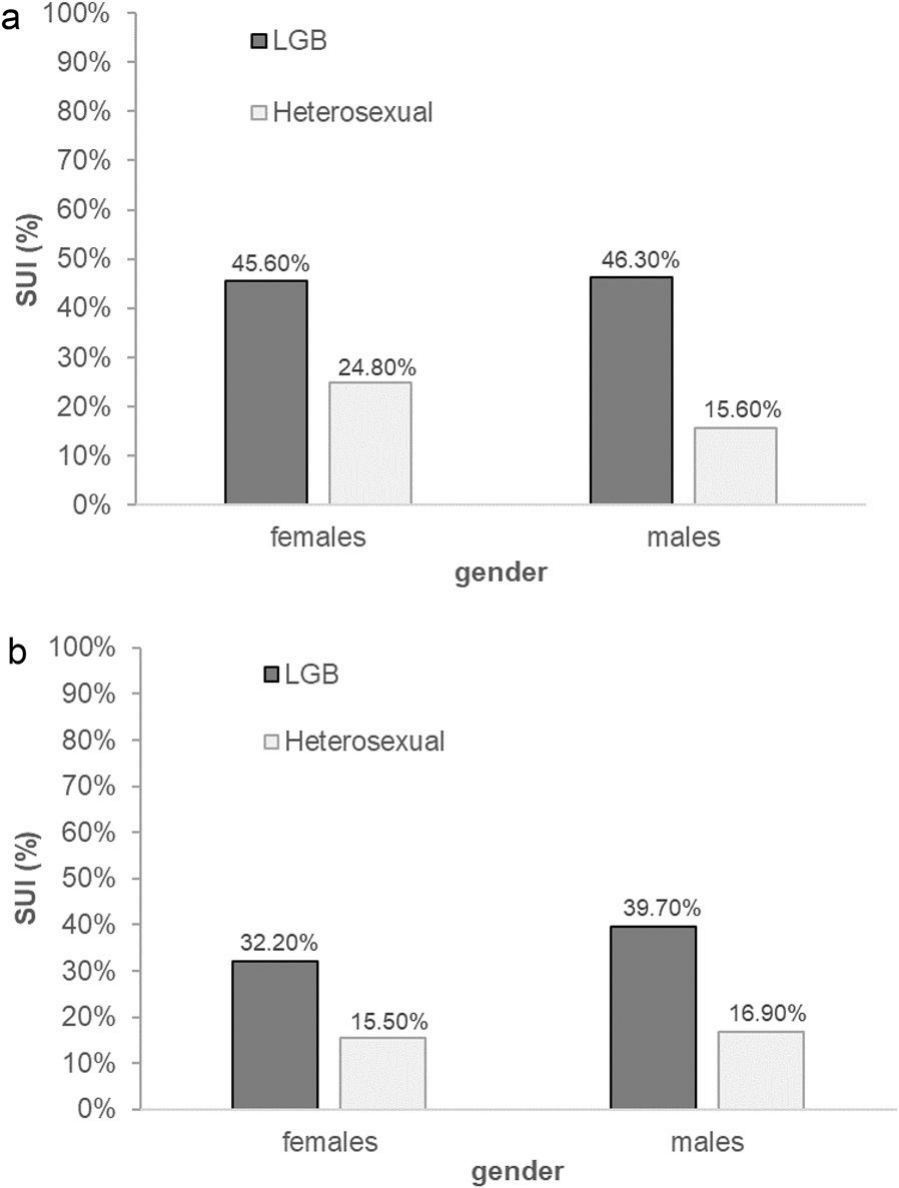
Percentages of suicidal ideation (SUI) in the past month in LGB and heterosexual female and male participants at *M* = 17.4 years of age (**a**) and *M* = 20.6 years of age (**b**). Statistical parameters of the chi-square tests. 17-year-old females: *X*^*2*^(1, *N* = 565) = 16.05, *p* < .001. 17-year-old males: *X*^*2*^(1, *N* = 542) = 24.42, *p* < .001. 20-year-old females: *X*^*2*^(1, *N* = 564) = 19.34, *p* < .001. 20-year-old males: *X*^*2*^(1, *N* = 542) = 19.56, *p* < .001

**Fig. 2:**
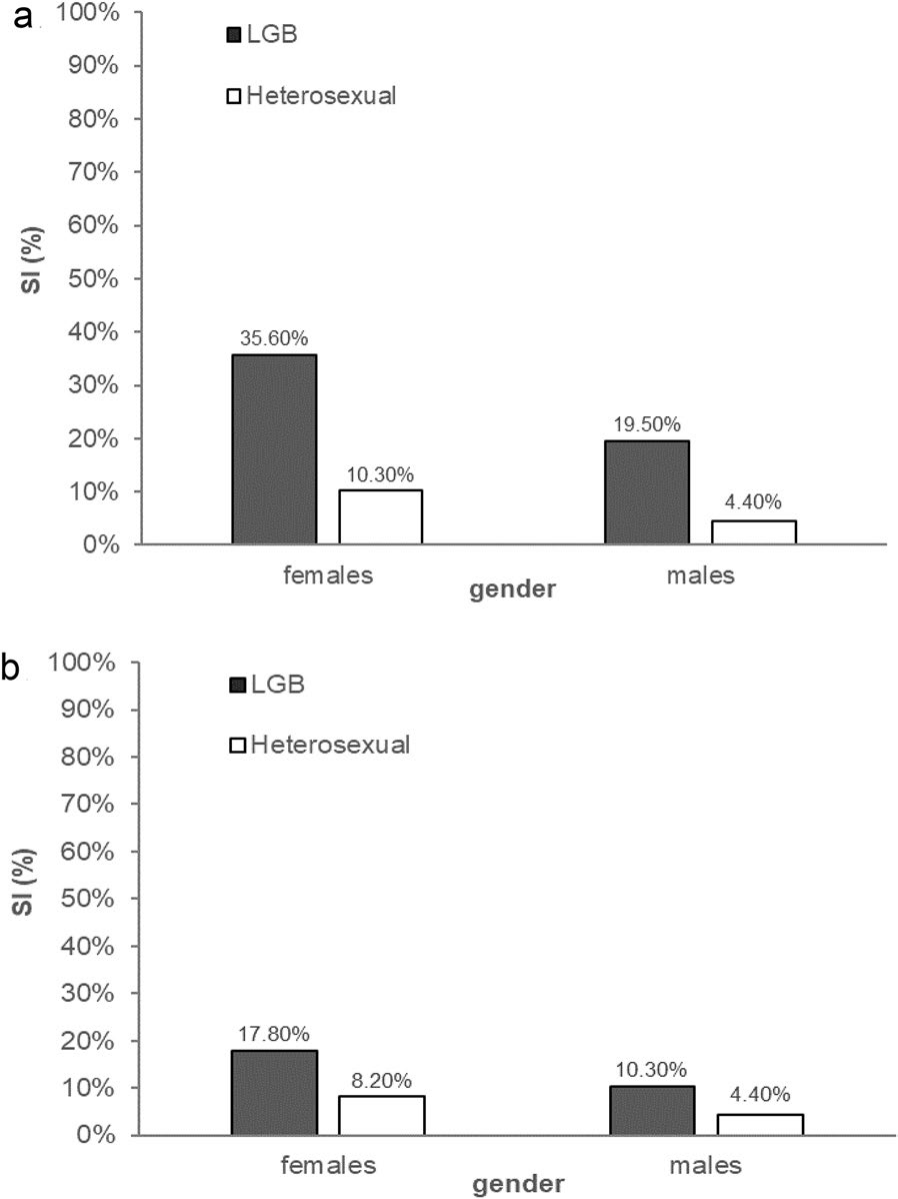
Percentages of self-injury (SI) in the past month in LGB and heterosexual female and male participants at *M* = 17.4 years of age (**a**) and at *M* = 20.6 years of age (**b**). Statistical parameters of the chi-square tests. 17-year-old females: *X*^*2*^(1, N = 566) = 39.39,* p* < .001. 17-year-old males: *X*^*2*^(1, *N* = 542) = 16.57, *p* < .001. 20-year-old females: *X*^*2*^(1, *N* = 566) = 10.55, *p* < .01. 20-year-old males: *X*^*2*^(1, *N* = 542) = 4.17, *p* < .05

**Fig. 3:**
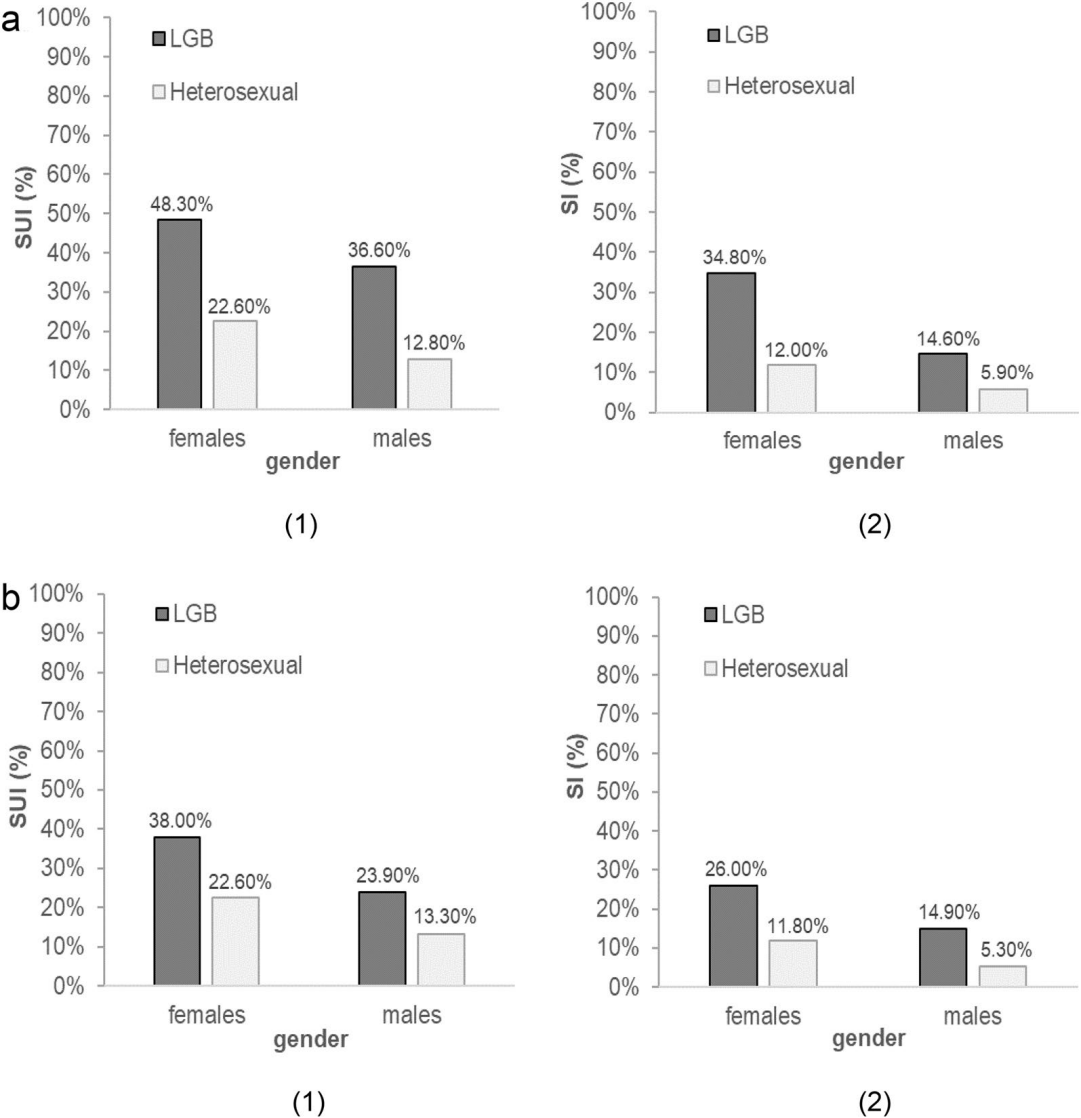
Percentages of suicidal ideation (SUI) (1) and self-injury (SI) (2) in the past month at the age of 15 years in LGB and heterosexual female and male participants at *M* = 17.4 years of age (**a**) and *M* = 20.6 years of age (**b**). Statistical parameters of the chi-square tests. Suicidal ideation (SUI): 17-year-old females: *X*^*2*^(1, *N* = 557) = 25.14, *p* < .001. 17-year-old males: *X*^*2*^(1, *N* = 534) = 17.20, *p* < .001. 20-year-old females: *X*^*2*^(1, *N* = 557) = 13.26, *p* < .001. 20-year-old males: *X*^*2*^(1, *N* = 534) = 5.28, *p* < .005. Self-injury (SI): 17-year-old females: *X*^*2*^(1, *N* = 556) = 29.55, *p* < .001. 17-year-old males: *X*^*2*^(1, *N* = 536) = 4.78, *p* < .05. 20-year-old females: *X*^*2*^(1, *N* = 556) = 16.17, *p* < .001. 20-year-old males: *X*^*2*^(1, *N* = 536) = 8.84, *p* < .01

Data were analysed using the statistical software IBM SPSS Version 24. Descriptive information regarding the study variables are presented in Tables 1 and 2. All variables of interest (SO, SUI and SI) were dichotomized to run chi-square tests (Figs. 1–3).

**Table 1:** Cross-tabulations of sexual orientation (LGB and heterosexual) for female participants (a) and male participants (b) at M = 17.4 years of age and at M = 20.6 years of age

(a) Females			
		20.6 years	
		LGB	Heterosexual
17.4 years	Heterosexual	14.10%	70.00%
	LGB	12.70%	3.20%

(b) Males			
		20.6 years	
		LGB	Heterosexual
17.4 years	Heterosexual	8.10%	84.40%
	LGB	4.40%	3.10%

**Table 2:** Frequencies of suicidal ideation (SUI) and self-injury (SI) (ever vs. never in the past month) for female and male participants at the three measurement waves (M = 15.4 years of age, M = 17.4 years of age and M = 20.6 years of age)

M = 15.4 years								
	Females				Males			
	Ever		Never		Ever		Never	
	n	%	n	%	n	%	n	%
SUI	149	26.8	408	73.2	78	14.6	456	85.4
SI	87	15.6	469	84.4	35	6.5	501	93.5

*M* = 17.4 years								
	Females				Males			
	Ever		Never		Ever		Never	
	n	%	n	%	n	%	n	%
SUI	159	28.1	406	71.9	97	17.9	445	82.1
SI	81	14.3	485	85.7	30	5.5	512	94.5

*M* = 20.6 years								
	Females				Males			
	Ever		Never		Ever		Never	
	n	%	n	%	n	%	n	%
SUI	113	20.0	451	80.0	107	19.7	435	80.3
SI	61	10.8	505	89.2	28	5.2	514	94.8

Ratings were provided on a 5-point Likert Scale ranging from never (1) to very often (5). Ratings were dichotomized for analyses (ever = 2–5 / never = 1). The past month was used as reference for the ratings

*Note 2*. Statistical parameters of the 2 (gender: female vs. male) × 3 (time-point: 15 years vs. 17 years vs. 20 yrs) mixed ANOVAs for SUI and SI:

SUI: sig. main effect of gender, *F*(1, 1087) = 16.62, *p* < .001, η^2^p = .02, with females (*M* = .25, *SD* = .43) reporting suicidal ideations more often than males (*M* = .17, *SD* = .38). Non-significant main effect of time-point, *F*(2, 2174) = 2.63, *p* = .07, η^2^p = .01. Significant interaction between gender and time-point, *F*(2, 2174) = 9.68, *p* < .001, η^2^p = .01. The follow-up pairwise comparisons revealed no significant effects, mean difference ≤ .01, *p*_s_ ≥ .06

SI: sig. main effect of gender, *F*(1, 1090) = 227.18, *p* < .001, η^2^p = .17, with females (*M* = .14, *SD* = .34) reporting self-injury more often than males (*M* = .06, *SD* = .23). Significant main effect of time-point, *F*(2, 2180) = 4.29, *p* < .05, η^2^p = .01, showing that self-injury at time-point 1 (15 years; *M* = .11, *SD* = .32) was significantly higher than at time-point 3 (20 years; *M* = .08, *SD* = .27). No significant interaction between gender and time-point resulted, *F*(2, 2180) = 1.69, *p* = .19, η^2^p = .01

## Results

### Sexual orientation (SO)

A total of 12.7% of the female participants (Table 1a) and 4.4% of the male participants (Table 1b) consistently reported identifying as LGB at 17 and 20 years of age. A total of 17.3% of females and 11.2% of males reported a change between ages 17 and 20 years (LGB to heterosexual attraction or vice versa). In both genders, an upward trend in reported bisexual and same-sex attraction across age was observed, with the increase being more pronounced in females.

### Suicidal ideation (SUI) and self-injury (SI)

Both genders reported a higher frequency of suicidal ideation in comparison to self-injury at all three measurement waves. In girls, reports of SUI increased from age 15 to 17 and decreased again at age 20. In boys, there was a steady increase in SUI from age 15 to 20. For SI, a continuous decrease between 15 and 20 years of age resulted in girls and boys [for more details see: 34].

### SO and the prevalence of SUI and SI

The prevalence of self-reported SUI (Fig. 1) and SI (Fig. 2) was significantly higher in male and female adolescents who reported identifying as LGB than in their heterosexual peers at 17 and 20 years of age. Except for SUI in 17-year-old girls, the prevalence of SUI and SI was more than twice as high as that in heterosexual peers. The results and statistical parameters for these chi-square tests are reported in Figs. 1 and 2.

### SO and the retrospective prevalence of SUI and SI

As information on LGB status was not available at the age of 15 years, we decided to run additional "retrospective" chi-square tests. To be able to make clearer statements regarding the relationships between SO, SUI and SI, only data from adolescents who had an LGB status at both study time points (ages 17 and 20 years) were analysed in relation to SUI and SI at the age of 15 years. LGB status was significantly associated with suicidal ideation at 15 years of age in 17-year-old and 20-year-old participants of both sexes. Similar significant associations resulted for LGB status at 17 and 20 years of age and self-injury at 15 years of age in female and male participants. The results and statistical parameters for these retrospective analyses are shown in Fig. 3.

### Gender, SO, SUI and SI

Looking at gender in general, girls reported higher frequencies for SUI and SI than their male peers at 15, 17 and 20 years of age (Table 2), regardless of sexual orientation (see Note 2 of Table 2 for statistical parameters of the mixed ANOVAs). These gender differences decreased with advancing age at the descriptive level. When additionally considering sexual orientation, gay and bisexual men reported SUI more often than lesbian and bisexual women did at both ages under study (Fig. 1). Lesbian and bisexual women, on the other hand, were more likely than their male counterparts were to report that they had injured themselves at both time points (Fig. 2). The same gender-related trends were observed in the heterosexual group, except for suicidal ideation at the age of 17 (female: 24.8% vs. male: 15.6%).

## Discussion

This is the first study from Switzerland to examine the correlations between aspects of suicidality and sexual orientation in adolescents using a longitudinal design. Similar to other findings, the results show that prevalence rates for suicidal ideation and self-injury in the current analyses were, with one exception, at least twice as high for adolescents identifying as LGB in comparison to their heterosexual peers, regardless of gender and age. Differences were significant at all time points under study, even though they decreased between 15 and 20 years of age on a descriptive level. Additionally, in line with previous studies [e.g., 33], gender differences in the prevalence of SUI and SI were found regardless of the SO.

A closer look at the results reveals that the differences in SUI and SI by LGB status were observed at ages 15, 17, and 20. This observation fits well with the idea that the development of one's own SO occurs by means of a multifaceted process at early ages in adolescence [35]. However, this unique situation can lead to intrapsychic tensions, especially for children and adolescents who perceive a sexual attraction, sexual preference or sexual identity outside the heteronormative conventions. At the age of 15 years, some adolescents might experience not only confusion about their SO but also proximal (e.g., feelings of shame and guilt) and/or distal (e.g., social exclusion and peer victimization) minority stressors, which in turn can lead to mental health problems and ultimately to self-harming behaviours [9]. This dynamic is likely to increase in cases where the SO formation process interacts with other salient personal, ethnic, cultural, and social identities [22] or, as in the case of bisexual individuals, break through the basic binary of the heteronormative matrix [23]. Our data coincide with cross-sectional findings from Switzerland [36] and point out in the same direction as other longitudinal studies, which emphasize that suicidal thoughts and behaviours are mediated by minority-specific stressors [37]. However, we cannot make any predictive statements regarding the SO and suicidality trajectories of the participants. Thus, future studies should address this problem by using more comprehensive and in-depth study designs, allowing for the analysis of the various trajectories of suicidal ideations, suicidal plans and different types of self-harming behaviours.

The trend that the associations of SO with SUI and SI become weaker with age corroborates data from previous studies that found the relative risk for suicide to be highest in 12- to 14-year-LGBT individuals [38]. After the age of 15 years, the suicide risk in the current sample decreased progressively on a descriptive level but remained higher than that in the heterosexual comparison group. This dynamic corresponds with the observation that in early adolescence, peer influence increases in terms of gender and sexuality-related norms, whereas in middle adolescence, the ability to stand up for oneself and escape peer pressure develops [39]. As a result, many LGB individuals complete the coming-out process during their college years [35]. Studies show that the actual coming-out is a critical situation that can have particularly negative consequences for mental health. These deleterious effects are mediated not only by SO but also by the quality of the environmental response to the coming-out message [12, 40]. However, it must be noted that the LGB community is not a uniform group, so that developmental trajectories of SO, including the timing and sequence of coming-out milestones, can vary considerably between individuals [41]. Consequently, the focus of future research should be on assessing intrapersonal, interpersonal and structural factors that allow a non-heterosexual identity to be developed safely.

Irrespective of age, females reported self-injuries more often than males did. This finding is consistent with a meta-analytical study that found nonsuicidal injuries to be more common in women [42]. Nevertheless, the same data contradict the studies that found suicidal acts more frequently in men [24]. Given that the distinction between suicidal and nonsuicidal acts is difficult and the question in our study included both aspects, a clear interpretation of this result is not possible. The situation regarding suicidal ideation is even more complex, as the gender ratios change from an initial female overrepresentation to a male overrepresentation in the LGB group at age 17 and in the heterosexual group at age 20. Even though the gender paradox observed in the LGB group seems to fit the fact that bisexual and gay men are more likely to question their sexual orientation than bisexual and lesbian women [43], no hasty interpretations should be made regarding these data. A more accurate interpretation would require in-depth research that considers the stratification by gender and sexuality in a more comprehensive manner.

## Limitations

The first limitation of the current research is its methodological nature. We decided to combine LGB orientation within one group for analyses. This decision was based on the surprisingly small numbers of individuals identifying as lesbian or gay at both ages and genders under study (0.54% and 1.26% of the sample, respectively), making reasonable statistical analyses impossible. In view of the expected frequency of LGB individuals (4.7% [3]), the question arises as to the factors that contributed to this difference in prevalence. One possibility would be that a disproportionately large number of people in the LGB group, especially lesbian and gay adolescents, decided not to take part in the survey. Another explanation would be that the participants feared opening to an unknown group of researchers about such a personal question as the one about the SO. Both hypotheses suggest that future surveys should be better designed and communicated, especially regarding the participation of sexual (and other) minorities.

Another limitation pertains to the measures used. Suicidal ideation and self-injury were assessed with only one item each. In future studies, it would be of interest to include an extended, more in-depth measure of suicidal ideation and self-injury to increase generalizability. Better differentiation between suicidal and nonsuicidal self-harm should also be introduced.

Sexual orientation is a complex, multidimensional construct incorporating the three dimensions of sexual attraction, sexual identity, and sexual behaviour. Because of its multifaceted structure, it is not easily quantified, measured and analysed. In accordance with Saewyc and colleagues [1], we assessed SO by means of sexual attraction at two measurement waves. However, same-sex attraction might overestimate the prevalence of LGB in youth, as same-sex attraction does not necessarily go along with self-identification as a member of the LGB community and same-sex behaviour [44]. Thus, future investigations would have to question SO more comprehensively. The distinction between gender identity and assigned gender would also have to be discussed to be able to make statements about young trans persons. In this context, the binary gender division should also be questioned, as it blocks the view of the situation of nonbinary young people.

Furthermore, current results are based on an urban sample of adolescents, which could therefore differ from findings obtained from adolescents living in areas that are more rural. Adolescents growing up in such rural areas might experience even greater pressure regarding their sexual orientation and coming-out process. It would be of interest to compare individuals of both areas regarding their coming-out process to gain more insights.

## Conclusions

The findings demonstrate the importance of acknowledging the fact that young people deal with their own SO at a very early stage in development. In many cases, the assumption of belonging to a sexual minority becomes a risk factor for the development of mental health issues. In terms of policy implications, these findings suggest that addressing both the reduction of minority stressors and the reinforcement of (inter)personal resources during secondary/high school may help to substantially reduce SO-based disparities in terms of mental health and suicidality.

School programs should enable age-appropriate but extensive sex education. During these educational hours, children/youth should be taught human diversity in terms of sexual orientation and their interactions with other axes of identity (e.g., gender identity). These interventions could help to change the school climate and reduce distal stressors that increase the risk of suicidal behaviours and suicide. In Switzerland, some initiatives have recently emerged in schools [45]. Nevertheless, there is a lack of national coordination and funding for these educational programs.

Additionally, it is of great relevance that teaching staff, paediatric health services, school psychologists and paediatric emergency room teams are made aware of the minority stress-related problems of LGB adolescents. If suicidal ideation and self-injury occur, it is important that these professionals talk promptly and with sufficient caution to the affected youth about their SO and the potential problems associated with it. A routine inquiry about sexual orientation in the clinical field would likely support not only the quality of the patient-practitioner relationship but also contribute to better mental health outcomes, especially in sexual minorities. However, the integration of this content in training programs of the different health professions rarely happens, which unfortunately perpetuates the structural stigmatization of this population group.

## Data Availability

The datasets used and analysed during the current study are available from the corresponding author on reasonable request.

## Abbreviations

LGB: Lesbian, gay and bisexual
SO: Sexual orientation
SUI: Suicidal ideation
SI: Self-injury
